# Effect of preoperative frailty on postoperative infectious complications and prognosis in patients with colorectal cancer: a propensity score matching study

**DOI:** 10.1186/s12957-024-03437-y

**Published:** 2024-06-12

**Authors:** Huipin Zhang, Hailin Zhang, Wei Wang, Yun Ye

**Affiliations:** 1 Department of Nursing, The First People’s Hospital of Changzhou and the 3rd Affiliated Hospital of Soochow University, 185 Juqian Street, Changzhou, Jiangsu 213000 China; 2 Department of Nursing, The Affiliated Lianyungang Hospital of Xuzhou Medical University, Lianyungang, Jiangsu 222061 China

**Keywords:** Frailty, Colorectal cancer, Complications, Adverse outcomes, Intra-abdominal infection, Propensity score matching

## Abstract

**Background:**

Few studies have explored the impact of preoperative frailty on infectious complications in patients with a diagnosis of colorectal cancer (CRC). Therefore, this study aimed to investigate the effect of preoperative frailty on postoperative infectious complications and prognosis in patients with CRC using propensity score matching (PSM).

**Methods:**

This prospective single-centre observational cohort study included 245 patients who underwent CRC surgery at the Department of Gastrointestinal Surgery, The Affiliated Lianyungang Hospital of Xuzhou Medical University between August 2021 to May 2023. Patients were categorised into two groups: frail and non-frail. They were matched for confounders and 1:1 closest matching was performed using PSM. Rates of infectious complications, intensive care unit (ICU) admission, 30-day mortality, and 90-day mortality, as well as postoperative length of hospital stay, total length of hospital stay, and hospital costs, were compared between the two groups. Binary logistic regression using data following PSM to explore independent factors for relevant outcome measures.

**Results:**

After PSM, each confounding factor was evenly distributed between groups, and 75 pairs of patients were successfully matched. The incidence of intra-abdominal infectious complications was significantly higher in the frail group than in the non-frail group (10.7% vs. 1.3%, *P* < 0.05). There were no significant differences in ICU admission rate, postoperative length of hospital stay, total length of hospital stay, hospital costs, 30-day mortality rate, or 90-day mortality rate between the two groups (*P* > 0.05). Our logistic regression analysis result showed that preoperative frailty (*OR* = 12.014; 95% *CI*: 1.334–108.197; *P* = 0.027) was an independent factor for intra-abdominal infection.

**Conclusions:**

The presence of preoperative frailty elevated the risk of postoperative intra-abdominal infectious complications in patients undergoing CRC surgery. Therefore, medical staff should assess preoperative frailty in patients with CRC early and provide targeted prehabilitation interventions.

## Background

According to a report by the World Health Organization, cancer ranks as the second leading cause of death worldwide [1]. Colorectal cancer (CRC) mortality is high among all cancer types. The global cancer statistics released by the International Agency for Research on Cancer in 2020 revealed that the incidence of CRC ranked second among all malignancies and fifth in terms of cancer-related mortalities in China [2], indicating that CRC poses a heavy burden on the health of the Chinese population.

Currently, there is no unanimous agreement on the definition of frailty. Fried et al. [3] proposed a frailty cycle model and defined frailty as a group of syndromes that leads to increased vulnerability and diminished stress resistance due to decreased physiological reserves or multisystem disorders of the body. This condition can lead to adverse clinical outcomes such as disability, falls, and fractures as a result of relatively small stressors such as minor infections and minor surgeries. In addition, frailty often overlaps with incapacity and chronic diseases, and patients are at a greater risk of disability and frailty, particularly when the chronic disease is cancer [4].

Surgery serves as one of the primary treatment modalities for patients diagnosed with CRC. Patients with CRC have a high prevalence of preoperative frailty due to the tumour’s hypermetabolic status, malnutrition due to gastrointestinal symptoms, and an inflammatory status [5]. Frailty assessment is a simple and accurate method for measuring the physiological reserves of patients and their ability to cope with surgical stress. It can help healthcare providers identify patients for whom surgery poses a high risk. Studies have shown that preoperative frailty in patients with CRC is associated with poor outcome measures, such as overall postoperative complications [6], severe complications [7], postoperative overall survival, and disease-free survival [8]. Postoperative infection is common in CRC patients. However, there is a paucity of studies exploring the impact of preoperative frailty on the occurrence of infectious complications.

In observational clinical study analyses, there are often many biases and confounding variables due to various reasons. PSM is a statistical method that can be used to analyse observational clinical study data [9]. It can circumvent the limitations of standard multivariate regression models effectively and reduce the effect of confounding variables, moreover, this method is not limited by the sample size [10].

Hence, the objective of this study was to assess the impact of preoperative frailty on postoperative infectious complications and other prognosis measures in patients with CRC, utilizing PSM.

## Methods

### Patients

This study is a prospective single-centre observational cohort study. Convenience sampling was used to select patients with CRC who underwent gastrointestinal surgery at the Affiliated Lianyungang Hospital of Xuzhou Medical University between August 2021 and May 2023 as the study participants. The inclusion criteria encompassed the following: (1) patients aged 18 years or older, (2) patients with a first pathological diagnosis of colon or rectal cancer, (3) those scheduled for elective radical surgery, and (4) patients with comprehensive clinical data. Conversely, the exclusion criteria were: (1) the presence of additional tumours or distant metastasis, (2) a history of mental illness, and (3) an inability to collaborate in the complete collection of data. The present study received approval from the Ethics Committee of the Affiliated Lianyungang Hospital of Xuzhou Medical University, with the ethics approval number KY-20211022023-01. It adhered to the ethical principles outlined in the Declaration of Helsinki, and informed consent was duly obtained from all participating patients.

### Measures

#### General patient information

Based on a literature review and the expertise of the investigators, the following information was obtained: sex, age, body mass index (BMI), albumin level, number of comorbidities, and tumour, node, and metastasis (TNM) stage.

#### Phenotype of frailty

This scale was proposed by Fried et al. [3] in 2001. It includes weight loss, slowness, exhaustion, low physical activity, and frailty. Each criterion was attributed 1 point. A score ≥ 3 points indicated frailty, 1 to 2 points indicated pre-frailty and 0 points indicated non-frailty. The short form of the International Short Physical Activity Questionnaire was used to assess the level of physical activity of each patient [11], and the evaluation criteria of the other indicators were based on the Fried phenotype cut-off value of the Taiwanese version [12]. In the current study, patients with ≥ 3 points were included in the frailty group and those with 0–2 points were included in the non-frailty group [13].

#### Outcome measures

The primary outcome measure focused on the occurrence of postoperative infectious complications. It mainly included intra-abdominal cavity, incision, urinary tract, pelvic cavity, and lung infections, with intra-abdominal infection encompassing anastomotic leakage [14]. Diagnostic criteria for infectious complications were defined according to previous literature and criteria as follows [15, 16]: (1) The diagnostic criteria for intra-abdominal or pelvic infection are the presence of any two of the following symptoms due to acute infection of abdominal or pelvic tissues: fever (≥ 38 °C), nausea and vomiting, abdominal pain, and jaundice, accompanied by abnormal laboratory and imaging findings, additionally, positive detection of pathogens or bacterial culture from the abdominal or pelvic contents is required; (2) The diagnostic criteria for incision infection are the presence of purulent discharge from the incision, pain, fever (≥ 38 °C), and other signs of infection, along with a positive culture of the incision secretion; (3) For urinary tract infection, the diagnostic criteria include frequent urination, urgent urination, dysuria, lower abdominal or renal pain, a white blood cell count in the urine of ≥ 5 per high-power field for males and ≥ 10 per high-power field for females, and positive urine culture results; (4) The clinical diagnostic criteria for pulmonary infection include symptoms such as cough, sputum, and moist lung rales, along with abnormal physical examination and imaging findings, and abnormal etiological examination results.

As for the secondary outcomes, they encompassed the rate of postoperative ICU admission, total length of hospital stay, postoperative length of hospital stay, hospital costs, 30-day mortality, and 90-day mortality.

### Data collection

Trained investigators conducted the study. General patient information and phenotype of frailty were obtained and assessed by the investigators on-site 1 day before surgery. TNM stage was obtained from postoperative pathology reports. Clinicians diagnosed postoperative infectious complications of patients according to the diagnostic criteria for nosocomial infections and documented them in an electronic medical record system. The remaining prognostic indicators were obtained using the hospital’s electronic information system and telephone follow-ups.

### Sample size

Since this study is observational, we utilised the sample size calculation formula for overall rate estimation: n = Z^2^_α/2_*P*(1-*P*)/δ^2^. Here, α is set at 0.05 (bilateral), corresponding to Z_0.05/2_ of 1.96. *P* represents the incidence of postoperative infectious complications among patients diagnosed with CRC. Based on our preliminary investigation of 20 patients, two (10.0%) developed such complications; thus, the *P* value was 0.10. The allowable error (δ) was set at 0.05. Based on these parameters, the calculated sample size was 138. To account for the potential ineffectiveness, we factored in a 20% increase, resulting in a minimum required sample size of 166 patients.

### Statistical analysis

All statistical analyses were conducted utilizing SPSS version 25.0 (SPSS Corp., Armonk, NY, United States). Normally distributed measurement data were expressed as mean ± SD. Measurement data with non-normal distribution were expressed as median and interquartile range. Enumeration data were expressed as frequencies and percentages. PSM was used for 1:1 closest matching with a calliper value of 0.03, and the patients were divided into groups according to whether or not they developed preoperative frailty. Statistical differences in confounding factors between the frail and non-frail groups before and after matching were analysed to determine whether matching was successful. The research indicates that the appropriate method for selecting covariates is to include confounding factors that are related to exposure and outcome [10]. A literature search indicated that sex [17, 18], BMI [17, 19], age [19, 20], albumin [21, 22], comorbidities [19, 23], and TNM stage [20, 24] have an impact on preoperative frailty and postoperative infectious complications, thus they were selected as covariates. The chi-square test, Fisher’s exact test, and Mann–Whitney U test were used to compare the differences in outcome measures between the frail and non-frail groups after PSM. If there were significant differences in outcome measures between the two groups, we conducted binary logistic regression using data before and after PSM to analyse factors influencing outcome measures. All tests were two-sided, and *P* < 0.05 was considered statistically significant.

## Results

### Baseline patient characteristics

A total of 270 questionnaires were distributed. Twenty-five patients met the exclusion criteria, including patients with other tumours or distant metastases known from postoperative pathology reports (eight patients), patients with suspended surgery (eight patients), patients who refused to cooperate with data collection (three patients), and patients lost to follow-up (six patients). After questionnaires from these excluded patients were removed, 245 valid questionnaires remained, representing an effective inclusion rate of 90.7%. Of the 245 included patients, 148 (60.41%) were male and 97 (39.59%) were female and the mean age was 67.15 years (range: 30–88 years). BMI values ranged from 14.02 to 35.76 kg/m^2^, with a mean of 23.73 kg/m^2^. Serum albumin levels varied from 26.4 to 47.5 g/L, averaging 36.8 g/L. The number of comorbidities ranged from 0 to 7, with a median of two comorbidities. Regarding the TNM stage, 55 patients (22.45%) were classified as stage I, 107 patients (43.67%) as stage II, and 83 patients (33.88%) as stage III.

### Preoperative frailty assessment

Patients in this study had a median frailty score of 3 (range: 0–5). Using the aforementioned dichotomisation method, 129 patients, accounting for 52.65% of the total, were classified into the frail group, whereas 116 patients, representing 47.35% of the total, were assigned to the non-frail group.

### Outcome measures

In the study, owing to the presence of multiple infectious complications among some patients, the aggregate count of infectious complications (24 cases) exceeded the number of patients who exhibited these complications (18 patients, representing 7.34% of the total study population). The frequency of different types of complications, in descending order, was as follows: intra-abdominal infection, 13 cases (5.30%); pelvic infection, three cases (1.22%); incision infection, three cases (1.22%), pulmonary infection, three cases (1.22%), and urinary tract infection, two cases (0.82%). Further details are listed in Table 1. In this study, 20 patients (8.16%) were admitted to the ICU, the median postoperative hospital stay was 14 days, the median total hospital stay was 19 days, the median hospital cost was 63,477 RMB, three patients (1.22%) died within 30 days after surgery, and four patients (1.63%) died within 90 days after surgery. Further details are presented in Table 2.

**Table 1:** Infectious complications in patients undergoing colorectal cancer surgery (*n* = 245)

Variables	Number (%)
Total infectious complications	18 (7.34)
Intra-abdominal infection	13 (5.30)
Pelvic infection	3 (1.22)
Incision infection	3 (1.22)
Lung infection	3 (1.22)
Urinary tract infection	2 (0.82)

**Table 2:** Prognostic indicators of patients undergoing colorectal cancer surgery (*n* = 245)

Variables	Number (%) / M (*P*_25_, *P*_75_)
ICU admission	20 (8.16)
Postoperative length of hospital stay (d)	14 (12, 17)
Total length of hospital stay (d)	19 (16, 23)
Hospital cost (RMB)	63,477 (58,643, 73,811)
Death within 30 d	3 (1.22)
Death within 90 d	4 (1.63)

### Comparison of postoperative characteristics before and after PSM

In this study, univariate analysis was conducted with the presence or absence of preoperative frailty serving as the dependent variable. The independent variables included sex, age, BMI, albumin level, the number of comorbidities, and TNM stage. The results before PSM revealed no statistically significant difference in sex or TNM stage between the frail and non-frail groups (*P* > 0.05). However, significant differences were observed in age, BMI, albumin level, and the number of comorbidities between the two groups (*P* < 0.05). These findings are summarized in Table 3 for further details.

To produce data suitable for between-group comparisons, PSM was used. Whether patients undergoing CRC surgery developed preoperative frailty was used as a grouping variable, and sex, age, BMI, albumin level, number of comorbidities, and TNM stage were used as covariates. PSM was performed for 116 patients in the non-frail group and 129 patients in the frail group.

The analysis revealed there were no significant differences in age, sex, BMI, albumin level, number of comorbidities, and TNM stage between the two groups after PSM (*P* > 0.05). Thus, each confounding factor was considered evenly distributed between the groups after PSM and 75 pairs of patients were successfully matched. Further details are presented in Table 4.

**Table 3:** Comparison of baseline data between the two groups before PSM (*n* = 245)

Variables	Non-frail (*n* = 116)	Frail (*n* = 129)	Statistical values	*P* value
Age, yr			12.249^a^	<0.001
< 60	38 (32.8)	18 (14.0)		
≥ 60	78 (67.2)	111 (86.0)		
Sex, *n* (%)			1.661^a^	0.197
Male	75 (64.7)	73 (56.6)		
Female	41 (35.3)	56 (43.4)		
BMI, kg/m^2^			-2.515^b^	0.012
< 18.5	6 (5.2)	11 (8.5)		
18.5–23.9	46 (39.7)	68 (52.7)		
24.0–27.9	51 (44.0)	40 (31.0)		
≥ 28.0	13 (11.2)	10 (7.8)		
Albumin, g/L			20.813^a^	<0.001
< 35	94 (81.0)	69 (53.5)		
≥ 35	22 (19.0)	60 (46.5)		
Comorbidities, *n* (%)			11.766^a^	0.001
0 ~ 1	62 (53.4)	41 (31.8)		
≥ 2	54 (46.6)	88 (68.2)		
TNM stage, *n* (%)			2.899^a^	0.089
I–II	83 (71.6)	79 (61.2)		
III	33 (28.4)	50 (38.8)		

a: Chi-square value; b: *Z* value; BMI: Body mass index; TNM: Tumour node metastasis

**Table 4:** Comparison of baseline data between the two groups after PSM (*n* = 150)

Variables	Non-frail (*n* = 75)	Frail (*n* = 75)	Statistical values	*P* value
Age, yr			0.350^a^	0.554
< 60	18 (24.0)	15 (20.0)		
≥ 60	57 (76.0)	60 (80.0)		
Sex, *n* (%)			2.199^a^	0.138
Male	47 (62.7)	38 (50.7)		
Female	28 (37.3)	37 (49.3)		
BMI, kg/m^2^			-0.216^b^	0.829
< 18.5	6 (8.0)	4 (5.3)		
18.5–23.9	34 (45.3)	38 (50.7)		
24.0–27.9	24 (32.0)	24 (32.0)		
≥ 28.0	11 (14.7)	9 (12.0)		
Albumin, g/L			0.141^a^	0.707
< 35	55 (73.3)	57 (76.0)		
≥ 35	20 (26.7)	18 (24.0)		
Comorbidities, *n* (%)			0.027^a^	0.869
0–1	31 (41.3)	32 (42.7)		
≥ 2	44 (58.7)	43 (57.3)		
TNM stage, *n* (%)			0.815^a^	0.367
I–II	51 (68.0)	56 (74.7)		
III	24 (32.0)	19 (25.3)		

a: Chi-square value; b: *Z* value; BMI: Body mass index; TNM: Tumour node metastasis

### C﻿omparison of outcome measures between the two groups after PSM

Analysis of post-PSM data from the non-frail and frail groups showed no significant differences in the incidence rates of total infectious complications, or pelvic, incision, pulmonary, and urinary tract infections (*P* > 0.05). Eight patients (10.7%) in the frailty group and one patient (1.3%) in the non-frailty group had an intra-abdominal infection, and the difference was statistically significant (*P* < 0.05). Further details are presented in Table 5. In terms of prognostic factors, analysis of post-PSM data from the non-frail and frail groups showed no significant differences in the ICU admission rate, postoperative length of hospital stay, total length of hospital stay, hospital costs, 30-day mortality, or 90-day mortality between the two groups (*P* > 0.05), as shown in Table 6.

**Table 5:** Comparison of infectious complications between the two groups after PSM (*n* = 150)

Variables	Non-frail (*n* = 75)	Frail (*n* = 75)	Statistical values	*P* value
Total infectious complications			2.836^a^	0.092
No	71 (94.7)	65 (86.7)		
Yes	4 (5.3)	10 (13.3)		
Intra-abdominal infection			5.792^b^	0.039
No	74 (98.7)	67 (89.3)		
Yes	1 (1.3)	8 (10.7)		
Pelvic infection			-	1.000^b^
No	73 (97.3)	74 (98.7)		
Yes	2 (2.7)	1 (1.3)		
Incision infection			< 0.001^c^	1.000
No	74 (98.7)	73 (97.3)		
Yes	1 (1.3)	2 (2.7)		
Lung infection			-	0.497^b^
No	75 (100.0)	73 (97.3)		
Yes	0 (0.0)	2 (2.7)		
Urinary tract infection			-	1.000^b^
No	75 (100.0)	74 (98.7)		
Yes	0 (0.0)	1 (1.3)		

a Chi-square value; b Fisher’s exact test, no chi-square value; c Continuity-corrected chi-square value.

**Table 6:** Comparison of prognostic indicators between the two groups after PSM (*n* = 150)

Variables	Non-frail (*n* = 75)	Frail (*n* = 75)	Statistical values	*P* value
ICU admission, *n* (%)			1.261^a^	0.262
No	70 (93.3)	66 (88.0)		
Yes	5 (6.7)	9 (12.0)		
Postoperative length of hospital stay, [d, *M* (*P*_25_, *P*_75_)]	14 (12, 18)	14 (12, 19)	-0.211^b^	0.833
Total length of hospital stay, [d, *M* (*P*_25_, *P*_75_)]	18 (16, 22)	19 (16, 24)	-0.441^b^	0.659
Hospital cost [RMB, *M* (*P*_25_, *P*_75_)]	62,653 (58,125, 71,131)	67,146 (58,681, 78,973)	-0.521^b^	0.602
Death within 30 d, *n* (%)			-	0.497^c^
No	75 (100.0)	73 (97.3)		
Yes	0 (0.0)	2 (2.7)		
Death within 90 d, *n* (%)			1.361^d^	0.243
No	75 (100.0)	72 (96.0)		
Yes	0 (0.0)	3 (4.0)		

a Chi-square value; b *Z* value; c Fisher’s exact test, no chi-square value; d Continuity-corrected chi-square value.

### Binary logistic regression analysis of risk factors for postoperative intra-abdominal infection

We performed binary logistic regression analysis of the data before and after PSM using the presence or absence of postoperative intra-abdominal infection as the dependent variable and age (< 60 years = 0, ≥ 60 years = 1), sex (male = 1, female = 2), BMI (< 18.5 kg/m^2^ = 1, 18.5–23.9 kg/m^2^ = 2, 24.0–27.9 kg/m^2^ = 3, ≥ 28.0 kg/m^2^ = 4), albumin (≥ 35 g/L = 0, < 35 g/L = 1), number of comorbidities (0–1 = 0, ≥ 2 = 1), TNM stage (I–II = 0, III = 1), and preoperative frailty (no = 0, yes = 1) as independent variables. Variables were entered into the equation at a level of 0.05 and eliminated at a level of 0.10. The results showed that BMI (*OR* = 2.913; 95% *CI*: 1.172–7.239; *P* = 0.021) and the number of comorbidities (*OR* = 0.156; 95% *CI*: 0.038–0.644; *P* = 0.010) were the influencing factors of intra-abdominal infection before PSM. Preoperative frailty (*OR* = 12.014; 95% *CI*: 1.334–108.197; *P* = 0.027) was a significant factor for intra-abdominal infection after PSM. See Table 7 for details.

**Table 7:** Binary logistic regression analysis of risk factors for postoperative intra-abdominal infection

Variables	Before PSM		After PSM	
	OR (95%CI)	*P* value	OR (95%CI)	*P* value
Age	6.330 (0.703–56.989)	0.100	2.658 (0.290–24.407)	0.387
Sex	0.221 (0.044–1.093)	0.064	0.379 (0.077–1.862)	0.232
BMI	2.913 (1.172–7.239)	0.021	2.276 (0.856–6.053)	0.099
Albumin	0.490 (0.115–2.093)	0.335	1.280 (0.261–6.271)	0.761
Comorbidities	0.156 (0.038–0.644)	0.010	0.417 (0.085–2.051)	0.282
TNM stage	0.352 (0.083–1.502)	0.158	1.261 (0.239–6.668)	0.785
Preoperative frailty	4.112 (0.978–17.287)	0.054	12.014 (1.334–108.197)	0.027

BMI: Body mass index; TNM: Tumour node metastasis

## Discussion

Numerous studies have demonstrated that preoperative frailty leads to an increased incidence of adverse postoperative outcomes in patients with CRC. To the best of our knowledge, this study marks the first attempt to establish a correlation between preoperative frailty and the occurrence of intra-abdominal infectious complications in patients diagnosed with CRC. This finding is encouraging, as it provides new avenues for exploring additional adverse outcomes associated with preoperative frailty.

The findings of this study revealed that the incidence of preoperative frailty among patients diagnosed with CRC was 52.65%, which is notably higher than that reported by McIsaac et al. [25] in their survey of patients undergoing non-cardiac surgery (3.1%). This disparity might stem from the fact that CRC lesions are situated within the digestive tract, leading to the prevalence of gastrointestinal symptoms such as constipation, diarrhoea, haematochezia, abdominal discomfort, and appetite loss. These symptoms affect a patient’s digestive and absorptive functions, leading to a reduced intake of nutrients, including carbohydrates, proteins, and vitamins, as well as an energy deficit. Furthermore, the hypermetabolic state associated with tumours further exacerbates the negative energy balance in patients, resulting in excessive breakdown of bodily muscles and adipose tissue, which in turn leads to decreased body mass and accelerates the onset of frailty. In addition, our results were higher than the preoperative frailty rate reported by Tsai et al. (22.7%) [26]. This difference may be attributed to variations in the frailty assessment tools employed as well as disparities in the geographical area, lifestyle, and dietary habits of the study populations. Despite significant variations in the reported prevalence of preoperative frailty across various studies, it remains at a high level and warrants serious attention from clinical staff.

The incidence of postoperative intra-abdominal infection in patients with CRC was the highest among all infectious complications (5.30%) in our study, and this was similar to the results of a previous study from Dominguez-Comesana et al. (5.8%) [27]. Another multicentre, large-sample prospective cohort study in China [14] reported an incidence of 7.25%, which was higher than the results of this study. In our study, the intra-abdominal infection included anastomotic leakage, as well as abscess and peritonitis. Anastomotic leakage has received attention in the literature as a potential outcome of CRC surgery. Mima et al. [20] discovered that preoperative frailty serves as an independent predictor for the occurrence of postoperative anastomotic leakage in patients with CRC after adjusting for age, tumour stage, and administration of adjuvant chemotherapy (OR = 1.77, 95% CI: 1.05–2.85; *P* = 0.032). Okabe et al. [28] assessed frailty using the Clinical Frailty Scale and found that frailty was associated with anastomotic leakage (*P* = 0.033). Both studies are similar to our findings, but it should be noted that although anastomotic leakage is a significant complication and an important subtype of intra-abdominal infection, it does not encompass the entire spectrum of such infections.

Of concern, our findings clearly indicate a relationship between preoperative frailty and an increased risk of intra-abdominal infectious complications in patients undergoing CRC surgery. Although the exact mechanism underlying this association remains unclear, several factors may contribute to this increased risk. Pei et al. [21] suggested that preoperative albumin level, lymphocyte/leukocyte ratio, subcutaneous fat mass, and skeletal muscle mass are associated with postoperative intra-abdominal infections in patients with CRC. Albumin, a marker of nutritional status, reflects the overall health and well-being of patients. A low lymphocyte/leukocyte ratio may indicate an inflammatory status [29], which may impact wound healing and increase the risk of infection. Low subcutaneous fat and skeletal muscle mass may indicate sarcopenia, which is associated with decreased immune function and reduced self-repair ability. Whereas as defined by Fried et al. [3], frailty includes multiple pathophysiological mechanisms, including malnutrition, inflammation, weight loss, and sarcopenia, which may have commonalities with the risk factors for intra-abdominal infection explored by Pei et al. [21]. These factors can have a negative impact on metabolism, bone mineral density, muscle mass and strength, exercise tolerance, and the vascular system [30]. This, in turn, may reduce the body’s capacity for fighting infections and repairing wounds, thereby increasing the risk of postoperative intra-abdominal infections. Moreover, frailty may lead to poor anastomotic healing, which can result in anastomotic leakage and subsequent intra-abdominal infection [31]. Oxidative stress, which involves the excessive production of reactive oxygen species, may play a crucial role in this process by contributing to the decline in muscle mass and function, promoting frailty, and hindering incision healing [32].

Therefore, identifying and addressing frailty in patients before CRC surgery is crucial to mitigate the risk of postoperative intra-abdominal infection. Future research should focus on elucidating the exact mechanisms underlying the association between frailty and intra-abdominal infections to develop targeted intervention strategies.

Our findings showed that preoperative frailty did not affect ICU admission rate, total length of hospital stay, postoperative length of hospital stay, hospital costs, 30-day, and 90-day mortality in CRC patients. However, some outcome measures occurred more frequently in the frailty group, including ICU admission rate (6.7% vs. 12.0%, *P* = 0.262) and 90-day mortality (0% vs. 4.0%, *P* = 0.243), which suggests that frailty may have a tendency to increase ICU admission rate and 90-day mortality in patients. Postoperative ICU admission may be related to factors such as unstable intraoperative and postoperative vital signs, while preoperative frailty will lead to decreased physical and psychological reserve of patients. When responding to the strong stressor of surgery, the body’s adaptability is weakened, making it easy to produce a serious and continuous stress response [3], and then affecting the vital signs of patients. Therefore, frailty may be a risk factor for increasing the ICU admission rate of patients. In addition, the study has shown that the 90-day mortality of frail patients is higher [25], which may be related to the reduced resistance and recovery of frail patients. This can increase the occurrence of complications such as postoperative infection in patients and further induce the failure of various organs in the body, thereby promoting the process of death.

Our study possesses several key strengths. Firstly, being a single-centre, observational, and prospective cohort study ensures the collection of comprehensive and accurate data. Furthermore, the surgical procedures and treatment protocols employed in our study were relatively standardised, minimizing the potential impact of confounding factors. Second, although intra-abdominal infection is a common complication in patients undergoing CRC surgery, previous studies have not given sufficient attention to the impact of preoperative frailty on the occurrence of intra-abdominal infections, and therefore, our study provides novel findings. However, it is important to acknowledge that our study also had some limitations. Our study population was small and only a few outcome measures were observed, which may have resulted in non-identification of statistical significance for some outcome measures. Second, our study population included only the Chinese population, and the conclusions obtained may not be extrapolated to other populations. Third, we included TNM stage as a covariate in this study. However, the specific T (tumour) and N (node) stages provide more detailed information compared to the overall TNM stage, and can more precisely reflect the local severity of the tumour. Finally, while we investigated CRC as a whole, a detailed classification of colon and rectal cancer could provide us with a clearer understanding of the respective incidences of infectious complications, as well as the impact of preoperative frailty on these complications.

## Conclusion

In conclusion, our results showed that preoperative frailty increased the incidence of postoperative intra-abdominal infectious complications in patients with CRC. Medical staff should pay attention to the preoperative frailty of patients and provide targeted prehabilitation interventions for patients with CRC. Future research should further explore the pathophysiological mechanism of preoperative frailty and intra-abdominal infectious complications.

## Data Availability

The datasets used and/or analyzed during the current study are available from the corresponding author on reasonable request.

## Declarations

### Reporting standards

The authors have read the STROBE guidelines and the manuscript has been prepared and revised accordingly to the STROBE guidelines.

## Abbreviations

BMI: body mass index
CRC: colorectal cancer
PSM: propensity score matching
